# The Cross-Talk Between the Dopaminergic and the Immune System Involved in Schizophrenia

**DOI:** 10.3389/fphar.2020.00394

**Published:** 2020-03-31

**Authors:** Pia M. Vidal, Rodrigo Pacheco

**Affiliations:** ^1^ Department of Basic Science, Biomedical Science Research Lab, Faculty of Medicine, Universidad Católica de la Santísima Concepción, Concepción, Chile; ^2^ Laboratorio de Neuroinmunología, Fundación Ciencia & Vida, Santiago, Chile; ^3^ Universidad San Sebastián, Santiago, Chile

**Keywords:** schizophrenia, dopamine receptors, T cells, microglia, peripheral monocytes, neuroimmunology, behavior

## Abstract

Dopamine is one of the neurotransmitters whose transmission is altered in a number of neural pathways in the brain of schizophrenic patients. Current evidence indicates that these alterations involve hyperactive dopaminergic transmission in mesolimbic areas, striatum, and hippocampus, whereas hypoactive dopaminergic transmission has been reported in the prefrontal cortex of schizophrenic patients. Consequently, schizophrenia is associated with several cognitive and behavioral alterations. Of note, the immune system has been found to collaborate with the central nervous system in a number of cognitive and behavioral functions, which are dysregulated in schizophrenia. Moreover, emerging evidence has associated schizophrenia and inflammation. Importantly, different lines of evidence have shown dopamine as a major regulator of inflammation. In this regard, dopamine might exert strong regulation in the activity, migration, differentiation, and proliferation of immune cells that have been shown to contribute to cognitive functions, including T-cells, microglial cells, and peripheral monocytes. Thereby, alterations in dopamine levels associated to schizophrenia might affect inflammatory response of immune cells and consequently some behavioral functions, including reference memory, learning, social behavior, and stress resilience. Altogether these findings support the involvement of an active cross-talk between the dopaminergic and immune systems in the physiopathology of schizophrenia. In this review we summarize, integrate, and discuss the current evidence indicating the involvement of an altered dopaminergic regulation of immunity in schizophrenia.

## Dysregulation of the Dopaminergic Neural Pathways in the Schizophrenia

Schizophrenia is a mental illness that often appears during late adolescence or early adulthood. It is characterized by thought disorders, perception, cognition and volition. The prevalence of this disorder reaches almost 1% of the world population, with an annual incidence ranging between 3.89 and 4.03 per 1,000 subjects (Moreno-Kustner et al., 2018). Its etiology is still unclear, and includes genetic and environmental components promoting alterations of dopaminergic signaling. The initial dopamine hypothesis stated that hyperactive dopaminergic transmission leads to development of schizophrenia symptoms (hallucinations, delusions, thought disorder, among others). However, several lines of evidence have shown that hypoactivity of frontal dopaminergic neurons in rodents (Pycock et al., 1980), non-human primates (Roberts et al., 1994), and humans (Ragland et al., 2007; Simpson et al., 2010) are also associated with schizophrenia. For instance, a pharmacological lesion of subcortical dopaminergic pathways in rats suggested a correlation between hyperactivation of subcortical dopaminergic neurons with hypoactivity of frontal dopaminergic neurons (Pycock et al., 1980). In addition, evidence obtained from humans has suggested that the polymorphism in the gene encoding catechol-O-methyltransferase, an enzyme involved in the degradation of dopamine, is associated with hypoactivity of prefrontal dopaminergic neurons in schizophrenia (Slifstein et al., 2008). Moreover, patients with frontal lobe damage as well as schizophrenia patients display similar alterations in the executive function (Ragland et al., 2007). Therefore, the current dopaminergic hypothesis involves hyperactive dopaminergic transmission in mesolimbic areas, striatum and hippocampus (Lodge and Grace, 2007; Patel et al., 2010; Weinstein et al., 2017), as well as hypoactive dopaminergic transmission in the prefrontal cortex of schizophrenic patients (Da Silva Alves et al., 2008). In addition, glutamatergic hypofunction has been suggested as one of the mechanisms involved in this dopaminergic dysfunction in schizophrenia (Swerdlow et al., 2009). In this regard, it has been hypothesized that DRD2-antagonism might prevent DRD1-mediated potentiation of N-Methyl-d-aspartate (NMDA) responses in the prefrontal cortex (Paz et al., 2008). Another line of evidence points to the changes in subcortical dopaminergic activity as one of the responsible circuits promoting alterations in glutamatergic neurotransmission in the substantia nigra (Mueller et al., 2004).

This, imbalance in the dopaminergic signaling has been differentially associated with the development of positive (presence of undesired cognitive/emotional functions, such as hallucinations, delusions, thought disorders, trouble concentrating, movement disorders) and negative (deficiency of desired cognitive/emotional effects, such as flattened affect, lack of pleasure, trouble with speech, apathy, concentration problems, and lack of motivation) symptoms. Positive symptoms have been related with stimulation of D2-like receptors, including DRD2, DRD3, and DRD4) (Li et al., 2016). Both primate and rodent brains express a higher density of D1-like (including DRD1 and DRD5) than D2-like receptors in healthy conditions (Weinstein et al., 2017). Meta-analysis of studies using positron emission tomography (PET) and single photon emission computed tomography (SPECT) have shown that presynaptic dopamine release is decreased in most brain regions of schizophrenic patients (Slifstein et al., 2015), except in the striatum, where the synthesis and the levels of dopamine released are increased (Mccutcheon et al., 2018; Avram et al., 2019). Furthermore, PET studies have demonstrated that prefrontal DRD1 expression is decreased in patients with schizophrenia (Kosaka et al., 2010), which has been associated with working memory deficits in the prefrontal cortex (Takahashi et al., 2008). In contrast, DRD1 expression is increased in the temporal and parietal cortex of schizophrenic patients, which might be associated with auditory hallucinations (Domyo et al., 2001). It has been described a moderate increase (10–20%) in the expression of DRD2 and DRD3 in the striatum of a subgroup of schizophrenic patients (Kestler et al., 2001). Moreover, DRD3 expression has been found to be enhanced in the basal ganglia, ventral forebrain (Gurevich et al., 1997), and blood lymphocytes of schizophrenic patients (Ilani et al., 2001). On the other hand, it has been shown that in comparison to healthy subjects, dopamine occupies a higher proportion of striatal D2-like receptors (Kegeles et al., 2010), and a bigger fraction of the dopamine transporters (DAT) in sensorimotor striatum (Weinstein et al., 2017) in schizophrenia.

Interestingly, it has been described a sub-regional heterogeneity in the dopaminergic dysregulation within the striatum. The greatest alterations in dopaminergic transmission have been observed in the associative striatum region. These alterations have been negatively correlated with verbal fluency performance in schizophrenic patients (Howes et al., 2009b). Since this brain region regulates information flow to and from the prefrontal cortex, the authors have suggested a potential link between striatal dopaminergic dysfunction and prefrontal alterations in schizophrenic patients (Howes et al., 2009b). A recent study showed that impaired connectivity between the cortico-striato-thalamo-cortical circuits is associated with cognitive difficulties in schizophrenic patients, including deficits in attention, memory, and executive function (Avram et al., 2018). Moreover, reduced striatal dopamine synthesis correlates with cognitive difficulties in patients during remission of positive symptoms, without an association with negative symptoms (Avram et al., 2019). Of note, the cohort of patients was taking antipsychotic drugs that did not seem to have a short-term clear effect on the results of the study (Avram et al., 2019). In addition, studies performed in schizophrenic patients have analyzed the expression levels of tyrosine hydroxylase (TH), the enzyme that catalyzes the first (and limiting) step in the biosynthesis of dopamine, and have found heterogeneous results, supporting either dopaminergic hyperactivity or hypoactivity (Akil et al., 2000; Mueller et al., 2004). One study has reported regional and laminar specific decrease of TH-immunoreactive axons in the entorhinal cortex of schizophrenic patients (Akil et al., 2000), whereas another study has shown increased TH mRNA levels in the dopaminergic neurons of the substantia nigra *pars compacta* of schizophrenic patients (Mueller et al., 2004).

Thus, current evidence indicates the involvement of complex alterations in the activity of neural dopaminergic pathways in the brain of schizophrenic patients, which are not completely consolidated. Therefore, further research is still needed to better understand the alterations of dopaminergic circuitry associated to the pathophysiological scenario of schizophrenia.

## Targeting the Dopaminergic System in Schizophrenia

The World Health Organization estimates that costs of schizophrenia in Western countries represent 1.6–2.6% of total health care budget, whereas in the US more than $60 billion USD per year are spent in this disorder (Howes et al., 2009a; Chong et al., 2016). The primary targets of many antipsychotic drugs for schizophrenia are striatal DRD2 and DRD3 (Howes et al., 2009a). However, the antagonism of these receptors is not always specific, and current drugs also act over other neurotransmitter receptors in the brain, including receptors for serotonin, histamine, norepinephrine, gamma-aminobutyric acid (GABA), and acetylcholine (Li et al., 2016). A DRD2 occupancy between 50% to 65%, is required in order to achieve clinical response to antipsychotic drugs and to minimize development of side-effects (e.g. extrapyramidal motor side effects) (Kapur et al., 2000). Targeting DRD2 using the antagonists chlorpromazine and haloperidol has been shown to effectively reduce positive symptoms, but ineffective at attenuating negative symptoms, cognitive deficits, and development of extrapyramidal motor side effects (Li et al., 2016). Of note, antipsychotic drugs might also increase the density of D2-like receptors in the striatum (Simpson et al., 2010). Antagonism of serotonin receptor 5-HT_2A_ in combination with DRD2-antagonism (e.g. clozapine and risperidone) have been shown to be more effective attenuating positive and negative symptoms, nevertheless, promoting the development of extrapyramidal motor side effects (Kinon and Lieberman, 1996) and others, such as gain of body weight, increase incidence of diabetes, loss of bladder control, and blurred vision (Snyder et al., 2015).

Moreover, patients who do not respond well to antipsychotic treatment have relatively normal levels of striatal dopamine compared with patients whose symptoms respond to antipsychotics (Demjaha et al., 2012). Treatment with the dopaminergic agonist apomorphine, ameliorates cognitive deficits and improves dopaminergic neurotransmission, which has been associated to the enhancement of prefrontal activity (Dolan et al., 1995), exaggerated stimulation of dopaminergic release, and potentially promoting more occupancy of D2-like receptors by dopamine in schizophrenic patients (Laruelle et al., 1996). On the other hand, the treatment with dopamine receptor blockers is more effective at ameliorating symptoms such as hallucinations or delusions. New antipsychotic drugs antagonizing preferentially DRD3 over DRD2 have shown cognitive performance improvement (Nakajima et al., 2013; Wang et al., 2017). However, further research is needed in this regard to fully understand how dopaminergic transmission, triggered through the stimulation of every single dopamine receptor subtypes, regulates the spectrum of positive, negative, and cognitive symptoms involved in schizophrenia. Of note, schizophrenia is a heterogeneous disorder and no single brain region or neurotransmitter is likely to explain all symptoms observed in all schizophrenic patients (Mccutcheon et al., 2019). Therefore, new drugs aiming to target beyond the dopaminergic system or involving modulation of multiple targets are more likely to effectively tackle positive and negative symptoms of schizophrenia. An example is the newer antipsychotic drug ITI-007, which is able to interact with the serotoninergic, dopaminergic, and glutamatergic pathways (Snyder et al., 2015). This drug has shown promising results either in safety and improving negative symptoms in a phase II randomized double-blind multicenter clinical trial (Lieberman et al., 2016).

The interaction between the dopaminergic and the immune system should also be considered for the development of new therapeutic targets in schizophrenia. In this regard, it is important to consider the role of tetrahydrobiopterin (BH4), which is an essential enzyme cofactor required for the production of tyrosine and dopamine (Felger et al., 2012). Some cytokines involved in inflammation might regulate the expression of GTP-cyclohydrolase I (GCH-1), the enzyme necessary for BH4 synthesis, thus increasing or decreasing dopamine biosynthesis rate. Nevertheless, inflammation may also increase reactive oxygen species and inducible nitric oxide synthase (NOS) activity, which lead to decreased BH4 availability and thereby reducing dopamine synthesis. For instance, the administration of Interferon alpha (IFN-α) in rats has been shown to promote a significant decrease in the levels of dopamine and BH4 in the amygdala and raphe area, an effect that was abolished upon administration of a NOS inhibitor (Kitagami et al., 2003). Similarly, IFN-α, Interleukin-6 (IL-6), and cardiotrophin-1, have also been shown to reduce the levels of BH4 in sympathetic neurons (Li et al., 2003). Conversely, IL-1β, IFN-γ, and TNF-α have been shown to increase BH4 synthesis, by inducing the expression and activity of GCH-1 in endothelial cells (Shi et al., 2004).

In addition to the effect in the biosynthesis of dopamine, some cytokines have shown to regulate dopamine storage in dopaminergic cells. In this regard, the pro-inflammatory cytokines IL-1β and TNF-α have been shown to decrease the expression of the vesicular monoamine transporter 2 (VMAT2), which is responsible for transporting cytosolic dopamine into secretory vesicles, and thereby limiting the availability of presynaptic dopamine. Conversely, TGF-α increases VMAT2 expression, favoring the storage of presynaptic dopamine (Kazumori et al., 2004). Taken together these results indicate that inflammatory cytokines exert a complex regulation in the levels of dopamine available in dopaminergic cells by modifying the biosynthesis rate and the storage of this neurotransmitter.

Adding another level of complexity in the interaction between the dopaminergic and immune system, some studies have shown that some drugs targeting dopaminergic system might regulate inflammation. According to the critical role of dopamine in the regulation of sepsis (Torres-Rosas et al., 2014), it has recently been shown that the antipsychotic drug trifluoperazine (TFP), which suppress dopamine secretion, exerted a strong regulation of pro-inflammatory cytokines and increased the survival rate in animal models of sepsis (Park et al., 2019). Another example illustrating the role of antipsychotic drugs is the study of the effect of paliperidone in neuroinflammation. The authors show that the pre-treatment of rats with paliperidone inhibited the stimulation of toll-like receptor 4 (TLR4) in a model of neuroinflammation induced by stress (Macdowell et al., 2014). In the same direction, another study has shown that haloperidol attenuates the activation of NF-κB, and consequently abrogated the production of pro-inflammatory cytokines in macrophages in response to lipopolysaccharide (LPS) (Yamamoto et al., 2016). Thus, these findings together illustrate how some antipsychotic drugs used for the treatment of schizophrenia might also induce anti-inflammatory effects by targeting the dopaminergic system in immune cells.

## Involvement of the Immune System in Cognitive Functions

Cognitive deficits in schizophrenia affect language, working and episodic memory, processing speed, stress resilience, social behavior, attention inhibition, and sensory processing (Kennedy and Adolphs, 2012; Brisch et al., 2014). Proper function of our cognitive and social abilities has partially been associated with the interaction between the central nervous system (CNS) and the immune system. The crosstalk between CNS and the peripheral cells is mediated by the glymphatic and meningeal lymphatic systems (Pape et al., 2019). In addition, the communication through the blood-brain barrier (BBB) and the endocrine system might also contribute to immunological alterations, affecting cognitive function. Here we analyze how some of these cognitive functions need the participation of the immune system.

### Memory and Learning

Immune cell infiltration has been considered a pathological hallmark of many CNS conditions. However, cells from either the innate and adaptive immune systems might exert beneficial effects in the CNS, as long as their recruitment and activation are well controlled. Cells from the immune system have been shown to play a role in spatial memory, learning, and neurogenesis (Kipnis et al., 2004b; Ziv et al., 2006).

The hippocampus is partially responsible for spatial learning/memory. Neurogenesis occurring in the hippocampal dentate gyrus has been shown to be dependent on the infiltration of mature CD4^+^ T-cells in the meninges (Ziv et al., 2006; Wolf et al., 2009), on the activation, phagocytic activity, and recruitment of microglia (Ziv et al., 2006), and on the increased production of brain-derived neurotrophic factor (BDNF) by glial cells (Wolf et al., 2009). Accordingly, the administration of minocycline, a non-specific anti-inflammatory drug, led to decreased neurogenesis in the dentate gyrus and abrogated the activation of meningeal of T-cells (Ziv et al., 2006; Derecki et al., 2010). Interestingly, a later study demonstrated that meningeal CD4^+^ T-cells participating in the acquisition of spatial memory and neurogenesis in the dentate gyrus are memory T-cells with specificity for self-antigens derived from the CNS (Baruch et al., 2013). Thus, these studies provide evidence that CNS-specific T-cells are required for neurogenesis, and their activation is dependent on microglial cells (Ziv et al., 2006; Derecki et al., 2010; Baruch et al., 2013).

Surgical removal of the deep cervical lymph nodes can result in dysregulated T-cell immunity that correlates with cognitive impairment (Radjavi et al., 2014b). Moreover, a decreased relative number of circulating dendritic cells, HLA-DR^+^ regulatory T-cells (Tregs), and CD4^+^ memory T-cells has been associated with more severe negative and cognitive symptoms in schizophrenic patients (Fernandez-Egea et al., 2016). According to the key role described for CNS-specific CD4^+^ T-cells in spatial memory, mice lacking peripheral mature T-cells manifest impaired spatial learning, memory capabilities (Kipnis et al., 2004b), and neurogenesis (Ziv et al., 2006) compared with the wild-type control group. This cognitive decline is reversed by transfer of T-cells (Kipnis et al., 2004b), but not when other immune cells (i.e. bone marrow-derived immune cells from T-cell depleted donors) are injected (Brynskikh et al., 2008). Similarly, it has been shown that the cognitive impairment developed by the deficiency of adaptive immune system is reversed just by the transfer of CD4^+^ T-cells, even in the absence of B-lymphocytes and CD8^+^ T-cells (Wolf et al., 2009; Radjavi et al., 2014b). Accordingly, a particular CD4^+^ T-lymphocyte subset, which originates from deep cervical lymph nodes (Radjavi et al., 2014a) and resides in the choroid plexus, ventricular margins, and subarachnoid spaces (Derecki et al., 2010; Baruch et al., 2013; Radjavi et al., 2014b) has been implicated in cognitive functions (Wolf et al., 2009; Radjavi et al., 2014b). These cells have been shown to decrease drug-induced psychosis and reduce cognitive impairment (Kipnis et al., 2004b) in mice. The training in spatial memory led to the accumulation of IL-4 producing T-cells in the meninges of experimental mice. Moreover, experiments in which the entrance of T-cells into the meningeal space was attenuated by using FTY720 or an anti-VLA4 antibody showed that reduced recruitment of a subset of memory CD4^+^ T-cells into the meninges resulted in impaired spatial memory (Derecki et al., 2010). However, these results do not rule out the indirect interaction of CD4^+^ T-cells with local or systemic antigen presenting cells (i.e. microglia, myeloid cells) and their secreted cytokines. For example, myeloid cells acquire an inflammatory phenotype in response to cognitive tasks in absence of meningeal T-cells. This phenotype can be reversed after injection of T-cells-expressing IL-4, which act on myeloid cells, favoring the acquisition of an anti-inflammatory phenotype (Derecki et al., 2010). These results suggest that T-cell derived IL-4 is the main cytokine regulating the phenotype of myeloid cells. Furthermore, peripheral macrophages alternatively activated *in vitro* in the presence of IL-4 acquire an anti-inflammatory phenotype that might improve learning and memory in the absence of CD4^+^ T-cells (Derecki et al., 2011).

Thus, these findings suggest that the key role of meningeal CD4^+^ T-cells in learning and memory is associated to their participation as a source of IL-4 in the brain. Interestingly, age-related cognitive impairment has been related with a shift on the regulatory cytokines produced in the choroid plexus, which constitute the entrance gate for CD4^+^ T-cells into the meninges. In this regard, high IL-4-to-IFN-γ ratio promotes CCL11 production, whereas low IL-4-to-IFN-γ ratio favors production of BDNF (Baruch et al., 2013). Remarkably, BDNF has been involved in neurogenesis and promoting learning and spatial memory (Derecki et al., 2010). Conversely, CCL11 is a chemokine associated with age-related cognitive impairments, whose high plasma levels correlate with reduced neurogenesis in mice and aging in humans (Villeda et al., 2011). These results highlight the importance of a cross-talk between meningeal lymphoid and myeloid cells for cognitive function. To add another piece to the puzzle, a recent study has raised new questions regarding the potential role of CD8^+^ T-cells derived IFN-γ from neurogenic brain niches in the generation of neurogenesis and cognition (Dulken et al., 2019). It has been shown in both rodents and humans that aging involves an increased CNS-infiltration of T-cells expressing IFN-γ, which decreases proliferation of neural stem cells (Dulken et al., 2019).

Importantly, another group of studies has provided evidence of a fundamental role of microglia in shaping neuronal circuitry by four different ways. First, by engulfing presynaptic termini in the healthy brain (Schafer et al., 2012; Meyer, 2013). Secondly, by limiting neurogenesis through the release of soluble factors, such as secretome after phagocytosis (Diaz-Aparicio et al., 2020), BDNF, insulin growth factor-1, TNF-α, pre-micro RNAs, among others (Rodriguez-Iglesias et al., 2019). Third, by inhibition of Sirt1/p65 signaling pathway in the dentate gyrus (Sellner et al., 2016). Fourth, by phagocytosis of apoptotic newborn cells in the dentate gyrus (Sierra et al., 2010). In this regard, the stimulation of the fractalkine receptor (CX3CR1) and the complement receptor 3 (CR3) signaling pathways have been shown to participate in these processes through the pruning of synaptic spines, engulfment of neurons during periods of active synaptic pruning (Schafer et al., 2012; Meyer, 2013). Indeed, the defective interaction between neurons and microglia given in *cx3cr1*-deficient animals leads to impaired synaptic maturation and reduced efficiency of synaptic transmission (Reshef et al., 2014; Basilico et al., 2019). Consequently, the deficiency in these signaling pathways results in neurological impairment. For instance, *cx3cr1*-deficient mice display impaired associative and spatial memory (Rogers et al., 2011), reduced neurogenesis in the dentate gyrus (Meyer, 2013), as well as increased levels of IL-1β in the hippocampus (Rogers et al., 2011). *Cx3cr1*-deficiency also leads to higher spine density, enhanced number of excitatory synapses (Meyer, 2013), and altered microglial morphology (Corona et al., 2010; Basilico et al., 2019) in comparison with wild-type controls. It has been suggested that one of the mechanisms involved in the learning deficits observed in *cx3cr1*-deficient mice is the inability to achieve long-term potentiation (LTP) in the hippocampus of these animals, which seems to be a consequence of the increased levels of IL-1β (Rogers et al., 2011; Liu et al., 2019). In addition, other studies have shown that CX3CL1 transiently potentiates NMDA-function, but inhibits hippocampal LTP, a process regulated through the stimulation of adenosine receptors (Maggi et al., 2009; Scianni et al., 2013). In the same direction, the pharmacological depletion of microglial cells mediated by the bilateral injection of clodronate into the dorsal hippocampus or by oral administration of PLX3397 showed alterations in spatial learning (Torres et al., 2016). Moreover, depletion of BDNF from microglial cells has been shown to reduce motor learning, recapitulating some of the behavioral alterations reported in microglia-depleted mice (Parkhurst et al., 2013). Regarding CR3, it has been shown that C3 deficiency leads to an enhanced learning and memory in mice when compared with their wild-type littermates (Shi et al., 2015; Shi et al., 2017). However, little is known regarding C3R deficiency in microglial cells. These findings together illustrate how the innate and adaptive immune response in the CNS play a relevant role in the proper development of neuronal circuitry and in cognitive tasks in healthy physiological conditions.

Finally, some studies have shown that BBB might play a relevant role modulating the immune response, thus affecting cognitive function in schizophrenia. For instance, increased expression of genes involved in immune function and inflammation have been detected in the choroid plexus of schizophrenic patients, which correlates with BBB permeability (Kim et al., 2016). On the other hand, decreased expression of the tight-junction protein claudin-5 at the BBB, correlates with impaired learning and memory, depression, anxiety, impaired social behavior, and altered locomotor activity in mice (Greene et al., 2018). Therefore, the BBB should also be considered as an important actor involved in the regulation of the cross-talk between immune system and the cognitive and behavioral impairment associated to schizophrenia.

### Stress Resilience

Another behavioral response that might be significantly regulated by immune cells, including T-cells, microglia, and peripheral monocytes, is the adaptation to psychological stress. In this regard, it has been shown that T-cell deficient mice, including severe combined immunodeficient (SCID) and nude mice, develop a worst adaptation than their immunocompetent counterpart in models of post-traumatic stress disorder (Cohen et al., 2006; Scheinert et al., 2016). Adoptive transfer of T-cells into either T-cell deficient (Cohen et al., 2006) or immunocompetent mice (Lewitus et al., 2008) improves the adaptation to psychological stress (Scheinert et al., 2016). Moreover, when immunodeficient mice where reconstituted with T-cells devoid of Tregs, psychological adaptation to stress was better than in mice replenished with the total T-cell compartment, containing both Tregs and effector T-cells (Teff) (Cohen et al., 2006). Further analysis of post-traumatic stress showed an association between lymphocyte recruitment into the choroid plexus and stress resilience, as well as increased hippocampal BDNF levels (Lewitus et al., 2008). Single-cell RNAseq analysis of T-cells recruited into the CNS upon psychological stress suggests a non-encephalitogenic origin, expressing *Foxp3*, *Gata3*, and Th2 genes (Kertser et al., 2019). It has been hypothesized that increased lymphocyte recruitment into the CNS might lead to the development of memory T-cells, that are necessary in order to promote homeostasis and enhance resilience to subsequent psychological stressful experiences (Lewitus and Schwartz, 2009). Accordingly, lymphocytes from chronically stressed mice adoptively transferred into immunodeficient mice (Rag2^−/−^) are able to confer anti-depressant like behavioral effects compared to the adoptive transfer of lymphocytes isolated from unstressed mice (Brachman et al., 2015; Scheinert et al., 2016). Thus, these findings support the hypothesis that psychological stress triggers the generation of memory T-cells, probably with specificity to CNS-derived self-antigens, which are recruited to the choroid plexus and confer resilience to future adverse psychological events.

Recent studies have shown that monocytes might also be involved in stress resilience. In a mouse model of severe psychological stress, leukocyte trafficking through choroid plexus was suppressed. The inhibition of glucocorticoid receptor signaling restored leukocyte trafficking through choroid plexus, which was associated to the recruitment of Th2 and Tregs cells into the CNS, leading to attenuation of post-traumatic behavioral deficit (Kertser et al., 2019). Accordingly, corticosterone mediated an increase of inflammatory circulating monocytes in mice behaviorally susceptible to stress (Niraula et al., 2018; Gururajan et al., 2019). It has been shown that peripheral monocytes might be recruited into the brain by microglial cells and their chemokines secreted (Weber et al., 2019), where they can exacerbate neuroinflammation (Niraula et al., 2018) and anxiety-like behavior (Wohleb et al., 2014). In addition, *cx3cr1*-deficiency in mice confers resilience to chronic unpredictable stress stimuli, thus suggesting a detrimental role of microglial cells in response to psychological stress (Hellwig et al., 2016; Rimmerman et al., 2017). In this regard, it has been shown that CX3CR1-signaling induces hyper-ramification of microglial cells in response to chronic stress, which was associated with the development of depressive-like behavior (Hellwig et al., 2016). Thus, the emerging evidence indicates that the innate and adaptive immune system play a fundamental role in the behavioral response to psychological stress.

### Social Behavior

Social behavior constitutes another response regulated by the immune system. In this regard, mice deficient in adaptive immunity display social deficits, as evidenced by anti-social behavior in validated behavioral tests of preference between another mouse or an inert object. This behavioral impairment has been attributed to the lack of IFN-γ production by meningeal T-cells. Accordingly, this deficit in social behavior might be reversed by administration of IFN-γ in the cerebrospinal fluid (CSF) or by the adoptive transfer of T-lymphocytes isolated from wild-type mice. The authors showed evidence indicating that IFN-γ stimulates GABAergic inhibitory neurons, triggering inhibitory neural circuits and thereby preventing hyper-excitability in the prefrontal cortex (Filiano et al., 2016). On the other hand, microglial cells have been reported to be involved in social behavior (Torres et al., 2016; Kopec et al., 2018), and to drive changes in affective behavior under exposure to psychosocial stress (Lehmann et al., 2019). In this regard, microglia abrogated the development of chronic social defeat-induced anxiety-like and antisocial behavior. Accordingly, microglia replenishment in the brain, just after psychosocial stress, leads to anxiety-like and antisocial behavior. A potential mechanism to explain the role of microglia in social behavior suggested by the authors involves the elevated reactive oxygen species produced by microglial cells during and after stress exposure (Lehmann et al., 2019). Another plausible explanation comes from a recent study involving the CSF-1/CSF-1R axis, which is required for development of most tissue macrophages including osteoclasts, brain microglial cells, and others. In this regard, it has been shown that the interference of the CSF-1/CSF-1R signaling pathway in cerebellar microglia leads to defective motor learning and impaired social interactions (Kana et al., 2019). This latter study is supported by another work that indicates a relevant role of the cerebellum as a regulator of major cognitive functions, such as expectations and reward (Wagner et al., 2017). Furthermore, it has recently been shown that the disruption of connectivity between the cerebellum and the right dorsolateral prefrontal cortex is associated with the severity of negative symptoms in schizophrenia (Brady et al., 2019). Another line of evidence shows microglial cells as key components organizing neuronal circuits involved in sex-associated social behavior during adolescence. In this regard, microglia and their complement-dependent phagocytosis promoted the elimination of D1-like receptors in the nucleus accumbens of male rats, a process that was required for natural developmental changes in male social play behavior (Kopec et al., 2018). Interestingly, a recent study provided evidence that this organized phagocytic process mediated by microglia and complement in the development of sex-associated social behavior during adolescence is promoted by the action of testosterone-induced endocannabinoids (Vanryzin et al., 2019).

Interestingly, maternal immune inflammation has also been linked to an increasing risk of developing schizophrenia in the progeny, where hippocampal microglial cells display an altered gene expression profile, including upregulation of genes involved in embryonic development, long-term neuronal plasticity, angiogenesis, and extracellular matrix organization, whereas genes involved in phagocytosis, cell migration, and inflammatory response were downregulated (Mattei et al., 2017). Thus, emerging evidence indicates that meningeal T-cells and microglial cells play relevant roles regulating social behavior.

## Dopaminergic Regulation of the Immune System

A number of catecholamine family members, including dopamine, have been extensively involved in the regulation of the immune response (Tracey, 2009), affecting both the innate and adaptive immune system (Pacheco et al., 2014; Vidal and Pacheco, 2019). In this regard, dopamine receptors are expressed on T and B lymphocytes, dendritic and NK cells, macrophages, microglia, intermediate monocytes, neutrophils, and eosinophils (Levite, 2016; Arce-Sillas et al., 2019). In this section we focused in the analysis of the dopaminergic regulation of T cells, microglia, and monocytes, since these immune cells have been implicated in cognitive functions.

### Dopaminergic Regulation of T Cells

The final outcome of dopamine effects on T-lymphocytes depends on dopamine concentrations, type of T-cells and activation status, as well as the dopamine receptors being expressed (Levite, 2016). In addition to the expression of dopamine receptors, the dopaminergic system in T-cells also involves some subsets of T-cells as sources of dopamine. For instance, human Tregs might synthesize and release dopamine, which exerts a negative feedback on their suppressive activity (Cosentino et al., 2007). More recently, another study revealed that follicular helper T (T_FH_) cells synthesize and store dopamine, which is released upon antigen-recognition to stimulate DRD1-signaling on B-cells as a costimulatory signal to induce antibody production (Papa et al., 2017).

A group of studies has addressed the effect of dopamine in Teffs and Tregs using pharmacologic approaches in experiments *in vitro*. These studies have shown that the stimulation of D1-like dopamine receptors favors the differentiation of naive CD4^+^ T-cells toward a Th2 phenotype (Nakano et al., 2009), and attenuates the regulatory activity of Tregs (Kipnis et al., 2004a; Cosentino et al., 2007). Pharmacologic evidence has also shown that DRD4-signaling induces T-cell quiescence (Sarkar et al., 2006). A recent study using genetic approaches revealed that DRD4-signaling in T-cells favors Th2 differentiation, promoting allergic asthma in newborn lungs (Wang et al., 2019). Addressing the role of DRD3-signaling in T-cells, pharmacologic and genetic evidence has recently shown that stimulation of this receptor potentiates T-cell activation, favoring Th1 differentiation and reciprocally dampening the acquisition of the Th2 phenotype, both *in vitro* and *in vivo* (Gonzalez et al., 2013; Franz et al., 2015; Contreras et al., 2016; Elgueta et al., 2019). In the same direction, pharmacologic evidence obtained with human T-cells has shown that DRD3-stimulation increases IFN-γ production and concomitantly decreases IL-4 and IL-10 release, along with and exacerbated expression of the activation marker CD25 (Ilani et al., 2004).

Mechanistic analyses carried out through pharmacologic and genetic approaches have shown that this DRD3-mediated potentiation of Th1-differentiation and concomitant repression of Th2-differentiation involves the upregulation of the regulatory protein SOCS5 (Contreras et al., 2016), as well as reduction of intracellular cyclic adenosine monophosphate (cAMP) levels and ERK phosphorylation (Franz et al., 2015). It is important to consider that, under chronic inflammatory conditions, DRD3-signaling in CD4^+^ T-cells also favors the expansion of the Th17-lienage (Contreras et al., 2016). Regarding DRD5-signaling in T-cells, pharmacologic and genetic evidence has shown DRD5-stimulation on CD4^+^ T-cells potentiates TCR-signaling (Franz et al., 2015) favoring a stronger T-cell activation *in vitro* and *in vivo* (Osorio-Barrios et al., 2018). Further analyses have shown that DRD5-signaling in Teff favors the acquisition of the Th17-lineage, whereas in Tregs increase the potency of their suppressive activity (Osorio-Barrios et al., 2018).

Despite that most studies addressing the dopaminergic regulation of T-cells have been focused in CD4^+^ T-cells, a few studies have also analyzed CD8^+^ T-cells. In this regard, a couple of studies using pharmacological approaches have shown that DRD3-stimulation on CD8^+^ T-cells potentiates IFN-γ transcription (Ilani et al., 2004), increases their integrin-mediated adhesion to fibronectin and intercellular adhesion molecule 1 (ICAM-1), and synergizes lymphocytes migration toward inflammatory chemokines (Watanabe et al., 2006). In addition, a recent study has shown that pharmacologic stimulation of D1-like dopamine receptors attenuates both the generation and the suppressive activity of regulatory CD8^+^ T-cells (Nasi et al., 2019). Taken together, these findings indicate that both CD8^+^ and CD4^+^ T-cells might undergo a complex dopaminergic regulation, which affects their activation, adhesion, migration, differentiation, and effector or suppressive function. Thus, alterations in physiological dopamine levels or the expression of dopamine receptors in T-lymphocytes, such as the case of schizophrenia, may exert strong changes in the behavior of T-cells.

### Dopaminergic Regulation of Microglial Cells

Microglial cells constitute a key cell of the innate immune response in the CNS, which play a central role in neuroinflammation (Gonzalez et al., 2015). Since microglial cells reside in the CNS, they are exposed to dopamine released by dopaminergic neural circuits of the CNS. In this regard, dopaminergic signaling has been reported to modulate different microglial functions, including their inflammatory activity (Yan et al., 2015; Dominguez-Meijide et al., 2017; Elgueta et al., 2017), migration (Farber et al., 2005), and cell adhesion (Fan et al., 2018). Accordingly, microglial cells express both dopamine D1-like and D2-like receptors (Farber et al., 2005; Huck et al., 2015). However, under inflammatory conditions the expression of dopamine receptors might strongly change (Huck et al., 2015). This is the case of the DRD2, whose expression is induced upon inflammatory stimulation of microglial cells following stroke (Huck et al., 2015). High dopamine levels attenuate the inflammatory activation of microglia by reducing the release of nitric oxide (Farber et al., 2005) and decreasing the extent of phagocytosis (Fan et al., 2018). It has been suggested that this process may be mediated by the stimulation of low-affinity dopamine receptors in these cells, including DRD1 and DRD2 (Dominguez-Meijide et al., 2017), leading to a reduction in the phosphorylation of ERK1/2 (Fan et al., 2018), and to the inhibition of the angiotensin type-1/NADPH-oxidase/superoxide axis (Dominguez-Meijide et al., 2017). In the same direction, it has been described that DRD1-signaling in microglial cells induces the cAMP-mediated degradation of the NLRP3 inflammasome, thus exerting a potent anti-inflammatory effect *in vivo* (Yan et al., 2015). Moreover, microglial complement-dependent signaling pathway mediates elimination of D1-like receptors in the nucleus accumbens of adolescent male, leading to the development of social behavior changes in male rats (Kopec et al., 2018). In addition, the stimulation of the DRD2 in homeostatic microglial cells has been shown to attenuate the inflammatory response (Dominguez-Meijide et al., 2017) by increasing p38MAPK and reducing the number of cellular processes (Fan et al., 2018). Furthermore, indirect mechanisms involving DRD2-signaling in astrocytes and triggering anti-inflammatory effects on microglial cells have been described. Accordingly, it has been shown that high-dopamine levels might exert down-regulation of angiotensin II release by astrocytes (Dominguez-Meijide et al., 2017), and also induce the upregulation of the anti-inflammatory molecule αβ-crystallin in astrocytes (Shao et al., 2013), thus attenuating inflammatory behavior in microglial cells.

On the other hand, emerging evidence has suggested that stimulation of high-affinity dopamine receptors in microglia promotes neuroinflammation. Accordingly, the treatment of primary cultures of activated microglia with a DRD2/DRD3 agonist, has been shown to increase the release of nitrite and IFN-γ (Huck et al., 2015). Moreover, inflammatory stimuli such as LPS, IFN-γ, or TNF-α, induced enhanced levels of intracellular BH4 in microglial cells, which promotes a higher rate of dopamine biosynthesis in mouse. A similar situation was observed in peripheral macrophages when stimulated by IFN-γ or LPS, which promoted the activity of the transcription factor NRF2 (Mcneill et al., 2015) and the consequent upregulation of GCH-1 in a rats (Sakai et al., 1995). Thereby, these findings suggest that pro-inflammatory stimuli in microglial cells promote a stronger capacity for dopamine biosynthesis in animal models. However, it is important to keep in mind that human cells are less efficient generating BH4 in comparison to other species (Schmidt et al., 2014).

Interestingly, dopaminergic regulation of microglial activity has also been studied under pathological conditions, such as those associated to amyotrophic lateral sclerosis (ALS) and Parkinson’s disease. For instance, the pharmacologic stimulation of DRD4 has been shown to suppress microglia recruitment to the site of inflammation, and thereby delaying the progression of ALS in a mouse model (Tanaka et al., 2008). Moreover, the systemic DRD3-antagonism has been shown to modulate the inflammatory response of astrocytes, attenuating microglial activation in the striatum in a mouse model of Parkinson’s disease (Elgueta et al., 2017; Elgueta et al., 2019). Taken together, the current evidence indicates that high-dopamine levels promote the stimulation of low-affinity dopamine receptors (including DRD1, DRD2, and DRD4), inducing an anti-inflammatory effect in microglia, while low-dopamine levels selectively stimulates high-affinity dopamine receptors (including DRD3 and DRD5), triggering inflammation, as proposed before (Pacheco, 2017).

### Dopaminergic Regulation of Monocytes

Under pathological conditions, such as those associated to neurodegenerative disorders (Gonzalez and Pacheco, 2014), or CNS injury (Wattananit et al., 2016; Olingy et al., 2017; Norden et al., 2019; Vidal et al., 2019) peripheral monocytes play an important role contributing to neuroinflammation. For instance, using a rat model of peripheral inflammation it has been shown that monocytes depletion, mediated by the peripheral administration of clodronate, mitigates the production of inflammatory mediators and microglial activation without affecting dopaminergic neuronal survival (Xie et al., 2017).

Similar to microglial cells, monocytes also express both dopamine D1-like and D2-like receptors (Mckenna et al., 2002; Coley et al., 2015). A few studies have addressed the role of dopaminergic signaling in monocytes function and have found that it is associated with migration, regulation of inflammatory mediators (Gaskill et al., 2012; Coley et al., 2015), and proliferation (Bergquist et al., 2000). In this regard, *in vitro* experiments using human monocytes have shown that high dopamine levels potentiate the production of IL-10 as well as CXCL8, while low dopamine levels favor the secretion of IL-6 and CCL-2, and decrease TNF-α production in response to LPS (Gaskill et al., 2012). In addition, it has been shown that dopaminergic signaling through D1-like dopamine receptors increases monocytes migration and adhesion (Coley et al., 2015). Interestingly, a recent study has shown that DAT hypofunction in mice, a condition associated with increased dopamine levels, correlates with reduced microglial activation and a lower extent of infiltration of monocyte-derived macrophages into the brain (Castellani et al., 2019), suggesting that high-dopamine levels attenuate neuroinflammation. Furthermore, it has been shown that high dopamine concentrations (10–100 µM) decrease proliferation of peripheral blood monocytes (Bergquist et al., 2000). In summary, the current evidence suggests that high dopamine levels, probably by stimulating DRD1 or DRD2 in monocytes (Pacheco et al., 2014), reduce the production of inflammatory mediators, proliferation and CNS recruitment, thus attenuating neuroinflammation. Conversely, the stimulation of high-affinity dopamine receptors in peripheral monocytes seems to favor the production of some inflammatory cytokines in these cells.

## Changes in Dopaminergic Regulation of the Immune System Associated to Schizophrenia

As described in section 3, the immune system plays an important role collaborating with the CNS to carry out some cognitive and behavioral function. Thereby, dysregulation of the immune system function might be involved in the development of neurologic diseases. In this regard, the association between psychiatric disorders, like schizophrenia, with altered immune responses has progressively gained interest over the past decade (Khandaker et al., 2015; Sellgren et al., 2019).

Several studies have reported immunological alterations associated to schizophrenia. In this regard, two meta-analysis have reported dynamic alterations in the profile of cytokine expression of schizophrenic patients depending on the stage of the disease (first episode versus relapsed patients) (Miller et al., 2011; Frydecka et al., 2018). These changes involve both inflammatory and anti-inflammatory cytokines depending on the disease duration, pharmacologic treatment, smoking status, among others (Miller et al., 2011; Muller et al., 2013). Another group of studies addressing gene expression in schizophrenic patients has suggested that dysregulation of the immune response associated to schizophrenia is a consequence of the disease progression or due to the long-term treatment with antipsychotic medication (Kumarasinghe et al., 2013; Frydecka et al., 2018).

**Figure 1:**
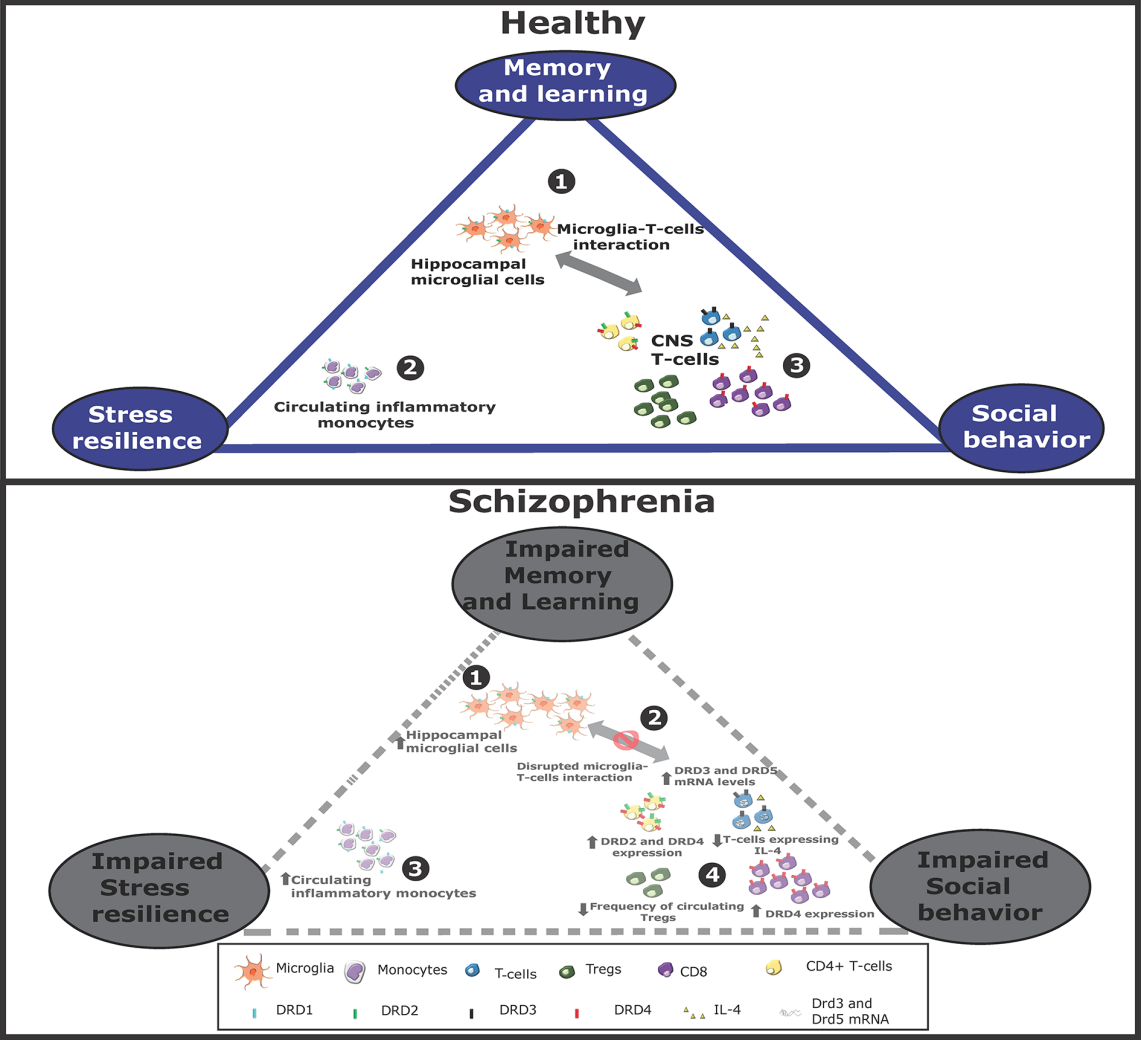
Altered dopaminergic signaling in monocytes, T-cells, and microglia associated to schizophrenia. Schematic representation of immune cells associated to cognitive/behavioral function in healthy conditions (top panel) or in schizophrenia (bottom panel). Top panel: In healthy conditions, 1. microglial cells stimulate to T-cells infiltrated into the CNS. 2. Peripheral monocytes might also infiltrate the brain and favor T-cell activation. 3. Activated T-cells contribute to stress resilience. Some T-cells acquire Th2 phenotype and produce IL-4, which favors neurogenesis and thereby memory and learning. Some T-cells acquire Th1 features, produce IFN-γ and promote social behavior. Bottom panel: In schizophrenia, 1. the interaction between microglia and T-cells is impaired, 2. thereby attenuating T-cell activation. Moreover, the expression of different dopamine receptors is altered, changing the extent of T-cell activation and differentiation. 3. Furthermore, the extent of peripheral monocytes and hippocampal microglia are increased, 4. whereas Treg frequency is reduced. All these changes hypothetically contribute to a reduced social behavior, decreased stress resilience and in impaired memory and learning.

A group of studies has analyzed samples of peripheral blood from patients and has found that dopamine receptors are differentially expressed in peripheral blood mononuclear cells (PBMCs) in schizophrenia. It has been reported that *DRD3* mRNA levels are increased in peripheral blood lymphocytes (Ilani et al., 2001). Consistently with the dopaminergic nature of drugs used in schizophrenia, it has been shown that pharmacological medication might induce changes in the expression of dopamine receptors in lymphocytes. In this regard, the transcriptional levels of DRD3 and DRD5 are increased in drug-free patients compared to medicated patients (Kwak et al., 2001). Moreover, the percentage of CD4^+^ and CD8^+^ T-cells expressing the DRD4, and CD4^+^ T-cells expressing DRD2 was increased in medicated schizophrenic patients compared to controls (Brito-Melo et al., 2012). Interestingly, two studies have reported changes in the relative composition of PBMCs associated to schizophrenia. The first one showed a higher proportion of circulating Tregs without changes in CD3^+^ or CD4^+^ T-cells in medicated-schizophrenic patients in comparison with healthy controls (Kelly et al., 2018). The second one, showed increased frequencies of NK cells, classical monocytes, naive B-cells, and CXCR5^+^ memory T-cells, and reduced percentages of dendritic cells, CD4^+^ memory T-cells, and HLA-DR^+^ regulatory T-cells in the blood of patients resistant to clozapine-treatment (Fernandez-Egea et al., 2016). Of note, the expression of DRD3 was shown significantly increased on peripheral CD4^+^ T-cells in clozapine-treated schizophrenic patients, which was correlated with reduced frequency of Tregs (Fernandez-Egea et al., 2016). According to this negative correlation, DRD3-signaling in CD4^+^ T-cells has been involved in promoting inflammatory responses, including Th1 and Th17 mediated immunity (Franz et al., 2015; Contreras et al., 2016). Following the same line, another study has shown an inverse correlation between the proportion of Tregs and the development of negative symptoms in schizophrenic patients (Kelly et al., 2018). Thus, the current evidence suggests an increased expression of high-affinity dopamine receptors, including DRD3 and DRD5, in T-lymphocytes of untreated schizophrenic patients, and enhanced levels of expression of the low-affinity dopamine receptor DRD2 in drug-treated schizophrenic patients. It is noteworthy that evidence obtained from *in vivo* approaches using animal models, or from *in vitro* approaches using human samples, has indicated that high affinity dopamine receptors DRD3 and DRD5 exert inflammatory effects while the low affinity dopamine receptor DRD2 promotes anti-inflammatory effects (Pacheco, 2017). Changes in dopaminergic regulation of the immune system associated to schizophrenia are integrated in **Figure 1**.

Interestingly, it has been shown that the treatment of schizophrenic patients with anti-inflammatory drugs in combination with anti-psychotic leads to better cognitive outcomes that the treatment with anti-psychotic drugs alone (Muller, 2010; Xiang et al., 2017). This is based on the inflammatory hypothesis of schizophrenia, which states that increased neuroinflammation contributes to the symptoms of schizophrenia. Therefore, combinatorial treatments using anti-inflammatory with anti-psychotic drugs might have synergistic effects at attenuating symptomatology, dampening the production of inflammatory cytokines, reactive oxygen species, prostaglandins and microglia function, among others (Sommer et al., 2014).

For instance, the treatment with the anti-inflammatory drug minocycline in combination with an atypical antipsychotic (risperidone, olanzapine, quetiapine, clozapine, or chlorpromazine), alleviates negative and positive symptoms in patients with schizophrenia (Levkovitz et al., 2010; Chaves et al., 2015). Of note, it has been suggested that minocycline acts not only by attenuating inflammation, but also decreasing synapse engulfment (Sellgren et al., 2019), and might potentially modify the composition of the gut-microbiota due to its antibiotic properties (Pape et al., 2019). The use of other anti-inflammatory therapies for schizophrenia are also being currently investigated in clinical trials, such as the treatment with monoclonal antibodies, such as Natalizumab, Tocilizumab, and Siltuximab (clinicaltrials.gov). It is noteworthy that a dopaminergic drug with anti-inflammatory activity is currently under study in humans as a treatment for schizophrenia. This is the case of a randomized, double-blind clinical trial ongoing in the US using a novel dopaminergic antagonist, I-tetrahydropalmatine, as an adjuvant treatment. This drug displays an anti-inflammatory activity and presents a higher affinity for D1-like receptors (clinicaltrials.gov).

## Immunological Alterations During Embryonic or Early Postnatal Development Leading to Imbalance in Dopaminergic Transmission and Neuropsychiatric Disorders in Adulthood

Diverse environmental risk factors associated with the development of schizophrenia during embryonic and early postnatal life have been reported, including perinatal hypoxia, cannabis consumption, stress, maternal infection, among others (Winter et al., 2009; Brown, 2011; Inta et al., 2011; Delpech et al., 2016). In the late 80’s a novel hypothesis for the development of schizophrenia was raised, which suggested that primary cerebral insults occur during early brain development, triggering disease manifestation years later (Meyer, 2013). The revised hypothesis incorporated to the first version the brain changes occurring during the early phases of the disease at the postnatal stage (Meyer, 2013). A study carried out in Denmark showed that both infections requiring hospitalization and autoimmune disease are risk factors for developing schizophrenia in the future (Benros et al., 2011). Moreover, maternal infection or immune activation during critical periods of pregnancy enhances the risk of the offspring to develop neuropsychiatric disorders later in life (Winter et al., 2009). Little is known regarding the molecular mechanisms explaining how a broad range of infectious agents might trigger schizophrenia. Indeed, the evidence suggests that a significant immune response in the mother during pregnancy is sufficient to trigger schizophrenia in the adult offspring, irrespective of the pathogen identity (Zuckerman and Weiner, 2005). To add another level of complexity, it has been shown that exposure to Epstein-Barr virus or throat infection in early childhood have also been associated with increasing risk of experiencing psychotic symptoms and/or subsequent neuropsychiatric disorders during the adolescence (Khandaker et al., 2014; Orlovska et al., 2017). Thus, the period of time in which infections can increase the risk of developing neuropsychiatric disorders is not restricted to the prenatal stage (Khandaker et al., 2012).

Some recent studies have demonstrated how maternal immune activation (MIA) during embryonic life might lead to changes in dopaminergic transmission during adulthood. In this regard, a preliminary study in a non-human primate model of MIA reported that male offspring born to MIA-treated dams had increased striatal dopamine in late adolescence compared with the control group (Bauman et al., 2019), changes similar to those associated with schizophrenia (see section 1). Similarly, prenatal exposure to the inflammatory agents Poly I:C or LPS in mice led to long-lasting changes in neurotransmitter levels and dopamine receptors in the brain of adult offspring. Specifically, levels of TH (Meyer et al., 2008), dopamine, and dopamine-derived metabolites were increased, whereas DRD1 and DRD2 (Meyer et al., 2008), serotonin receptors, and its metabolites were reduced in some specific brain areas (Winter et al., 2009). Moreover, organotypic cultures of ventral mesencephalon and striatum of rat fetuses exposed to LPS at embryonic day (E) E10, E14, or E18 showed a reduction in dopaminergic neurons when the cultures were kept for a long period compared with organotypic cultures of non-exposed fetuses (Snyder-Keller and Stark, 2008). In addition, prenatal exposure to inflammation also induces changes in pre- and post- synaptic GABAergic, glutamatergic, and serotoninergic neuronal circuits. Changes associated to embryonic exposure to inflammatory stimuli are not limited to neurons, but also to glial cells affecting the number, structure, positioning, and survival of these cells, as well as morphological changes in the brain (Boksa, 2010; Chua et al., 2012). With regard to these latter analyses, controversial results have been reported about the effect of embryonic exposure to inflammation in microglial density, proliferation, phagocytic activity, and number of cells in animal models (Boksa, 2010). Some studies have reported an increase in these parameters (Juckel et al., 2011; Hadar et al., 2017) while others have reported no change (Garay et al., 2013; Mattei et al., 2014). This discrepancy can mainly be attributed to the rodent strain used and the stage of gestation where pregnant mothers receive the injection of Poly I:C (Juckel et al., 2011; Garay et al., 2013; Mattei et al., 2014). Of note, although alterations in the number and morphology of microglia cells may be a transient process, they might lead to long-lasting changes at the gene expression level increasing, for example, their phagocytic activity (Delpech et al., 2016; Mattei et al., 2017), and acquiring a “primed” pro-inflammatory phenotype prone to neuroinflammation in the adulthood (Norden et al., 2015). Microglia isolated from mice that underwent maternal immune activation in *utero* have shown altered expression of genes involved in embryonic development and phagocytosis (Mattei et al., 2017), which resemble those alterations observed upon interruption of the CSF-1/CSF-1R axis in cerebellar microglia associated with motor and social deficits (Kana et al., 2019). Intriguingly, it has been shown that cesarean section births also induce long-term changes in brain dopamine receptors, specially D1-like receptors (El-Khodor and Boksa, 2001). Thus, current evidence suggests that inflammatory events occurring during embryonic or early postnatal age lead to alterations in microglial cells which later in life result in imbalance of dopaminergic transmission and the consequent development of neuropsychiatric disorders.

Anti-inflammatory treatments have shown to partially attenuate or even fully prevent the development of schizophrenia in animal models. For instance, minocycline treatment in the offspring reduced the levels of TNF-α and IL-1β in the hippocampus of rats exposed to MIA (Mattei et al., 2014). Furthermore, the authors showed that the treatment with minocycline promoted neurogenesis, improved working memory, and social behavior as well as increased the phagocytic activity of microglia in the hippocampus (Mattei et al., 2017). In addition, it was shown that deep brain electrical stimulation treatment during the adolescence of MIA rats prevented the behavioral impairment and attenuated the increase of microglial density (Hadar et al., 2017). An important consequence of LPS-induced MIA is the induction of metallothionein, a zinc-binding protein, in the mother’s liver, which promotes fetal zinc deficiency. Accordingly, maternal dietary zinc supplementation has been assessed as a treatment for offspring exposed to MIA. In this regard, it has been shown that zinc supplementation prevented the development of long-term cognitive abnormalities, reduced the number of TNF-α^+^ cells, and decreased the extent of apoptotic cells in the offspring of LPS-treated mice (Coyle et al., 2009; Chua et al., 2012). Thus, prophylactic anti-inflammatory treatments intended to target maternal inflammation in experimental animal models of schizophrenia have sparked interest in the combinatorial use of anti-inflammatory drugs with atypical antipsychotic drugs during the early phases of schizophrenia. Preliminary studies in human patients with schizophrenia have shown promising beneficial effects by improving negative and cognitive symptoms compared with patients receiving antipsychotic drugs alone (Levkovitz et al., 2010; Muller et al., 2010).

## Conclusions

Schizophrenia is associated with dysregulated activity of dopaminergic neural circuits, which consequently promotes several cognitive and behavioral alterations. Immune system cells, specially T-cells, microglial cells and peripheral monocytes, have been described to collaborate with the CNS to carry out some of these cognitive and behavioral functions that are altered in schizophrenia. Furthermore, dopamine might strongly affect the activity of T-cells, microglia, and peripheral monocytes, since all these immune cells express dopamine receptors. The current evidence indicates that high-dopamine levels promote the stimulation of low-affinity dopamine receptors (including DRD1, DRD2, and DRD4), inducing an anti-inflammatory effect on immune cells, while low-dopamine levels selectively stimulates high-affinity dopamine receptors (including DRD3 and DRD5), triggering inflammation. Thus, alterations in dopamine levels associated to schizophrenia might affect inflammatory response of immune cells and consequently some behavioral functions, including reference memory, learning, social behavior, and stress resilience (see a summary in **Table 1**). Interestingly, studies performed with patients have shown that drug-free schizophrenic patients display exacerbated expression of high-affinity dopamine receptors, and thereby acquiring pro-inflammatory features in response to dopamine. Conversely, medicated patients seem to switch their expression of dopamine receptors favoring the expression of low-affinity receptors in immune cells, thus acquiring anti-inflammatory profiles in response to dopamine. Accordingly, recent data obtained from clinical trials has suggested that the usage of anti-inflammatory and/or dopaminergic drugs as adjuvant therapy for schizophrenia might give a significant improvement in the symptomatology involved in this disorder. Recent evidence indicates that significant inflammatory events occurring during embryonic or early postnatal age lead to alterations in microglial cells which later in life result in imbalance of dopaminergic transmission and the consequent development of neuropsychiatric disorders. Further research is necessary in this area to decipher the molecular and cellular underlying mechanisms and, consequently, to be able to design therapeutic strategies to target specific detrimental processes without affecting the general function of the immune system. Studies about the long-term safety of combined therapies (i.e. anti-psychotics with anti-inflammatory drugs) are also still necessaries, as chronic administration of anti-inflammatory drugs could yield hepatic failure and/or an immunosuppressive state that may be involve increased susceptibility to infections and cancer. Finally, another aspect that should be further explored in the upcoming future is the validation of the cross-talk between immune system and CNS in the development of cognitive and behavioral changes, as most research in this field has been done in animal models.

**Table 1:** Dopaminergic regulation of the immune system associated with cognitive/behavioral functions.

Immune cells	Cognitive/behavioral function	Physiological effect involved	Dopaminergic regulation	Dopaminergic alteration in schizophrenia
**CD4^+^ T-cells and microglial cells**	Memory and learning (Ziv et al., 2006; Derecki et al., 2010; Baruch et al., 2013).	Neurogenesis.	Stimulation of D1-like dopamine receptors promotes the differentiation of naive CD4^+^ T-cells toward a Th2 phenotype (Nakano et al., 2009).	Increased *DRD3 and DRD5* mRNA levels peripheral blood T-cells (Ilani et al., 2001; Kwak et al., 2001).
		Adaptation to psychological stress.	Stimulation of D1-like dopamine receptors attenuates the regulatory activity of Tregs (Kipnis et al., 2004a; Cosentino et al., 2007).	Increased percentage of CD4^+^ T-cells expressing the DRD4 and DRD2 in medicated schizophrenic patients (Brito-Melo et al., 2012).
**T-cells and Tregs**	Stress resilience (Cohen et al., 2006; Lewitus and Schwartz, 2009; Scheinert et al., 2016).	Production of IFN-γ that stimulates GABAergic inhibitory neurons.	DRD3 signaling favors Th1 and inhibits Th2 differentiation (Gonzalez et al., 2013; Franz et al., 2015; Contreras et al., 2016).	Reduced percentage of Tregs in schizophrenic patients (Fernandez-Egea et al., 2016).
			DRD4 signaling induces T-cell quiescence (Sarkar et al., 2006).	
**T-cells**	Social behavior (Filiano et al., 2016)		DRD4 signaling promotes Th2 differentiation (Wang et al., 2019).
**CD8^+^ T-cells**	Memory and learning (Dulken et al., 2019),	Neurogenesis and cognition.	DRD3 signaling promotes IFN-γ (Ilani et al., 2004) transcription, and lymphocytes migration (Watanabe et al., 2006).	Increased percentage of CD8^+^ T-cells expressing DRD4 in medicated schizophrenic patients compared to controls (Brito-Melo et al., 2012).
			D1-like dopamine receptor signaling attenuates both the generation and the suppressive activity of regulatory CD8^+^ T-cells (Nasi et al., 2019).	
**Microglial cells**	Memory and learning (Rogers et al., 2011; Schafer et al., 2012; Meyer, 2013).	Synaptic pruning.	Upregulation of DRD2 upon inflammation (Huck et al., 2015).	Increased hippocampal microglial cells (Busse et al., 2012).
			Attenuation of inflammatory activation of microglial cells at high dopamine levels (Farber et al., 2005; Yan et al., 2015).	
**Microglial cells**	Social behavior (Kana et al., 2019; Lehmann et al., 2019).	High production of reactive oxygen species under stress, interference of the CSF-1/CSF-1R signaling pathway.	DRD3-antagonism in astrocytes dampens inflammatory features of microglial (Elgueta et al., 2017; Montoya et al., 2019).	
**Monocytes, Th2 and microglial cells**	Stress resilience (Kertser et al., 2019; Weber et al., 2019).	Trafficking of immune cells, production of cytokines.	High dopamine levels promote an anti-inflammatory phenotype (Gaskill et al., 2012).	Increased frequency of circulating inflammatory monocytes (Mckim et al., 2018).
			D1-like dopamine receptor stimulation increases monocytes migration (Coley et al., 2015).	

## Author Contributions 

Both authors contributed in analyzing the bibliography and writing the paper.

## Funding

This work was supported by *Programa de Apoyo a Centros con Financiamiento Basal* AFB-170004 (to Fundación Ciencia & Vida) from “Comisión Nacional de Investigación Científica y Tecnológica de Chile (CONICYT)” and by grants FONDECYT-1170093 (to RP) from “Fondo Nacional de Desarrollo Científico y Tecnológico de Chile” and the FAA 02/2019 from Universidad Católica de la Santísima Concepción (to PV).

## Conflict of Interest

The authors declare that the research was conducted in the absence of any commercial or financial relationships that could be construed as a potential conflict of interest.
